# Prognostic Value of Myeloid Differentiation Primary Response 88 and Toll-Like Receptor 4 in Breast Cancer Patients

**DOI:** 10.1371/journal.pone.0111639

**Published:** 2014-10-31

**Authors:** Fang-Jing Ma, Zhe-Bin Liu, Xin Hu, Hong Ling, Shan Li, Jiong Wu, Zhi-Ming Shao

**Affiliations:** 1 Department of Breast Surgery, Key Laboratory of Breast Cancer in Shanghai, Fudan University Shanghai Cancer Center, Shanghai, P.R. China; 2 Department of Oncology, Shanghai Medical College, Fudan University, Shanghai, P.R. China; 3 Department of Breast Surgery, the First Affiliated Hospital of Xinjiang Medical University, Urumqi, Xinjiang Uygur Autonomous Region, P.R. China; University of South Alabama, United States of America

## Abstract

**Purpose:**

Breast cancer remains a major cause of death in women worldwide, and tumor metastasis is the leading cause of death in breast cancer patients after conventional treatment. Chronic inflammation is often related to the occurrence and growth of various malignancies. This study evaluated the prognosis of breast cancer patients based on contributors to the innate immune response: myeloid differentiation primary response 88 (MyD88) and Toll-like receptor 4 (TLR4).

**Methods:**

We analyzed data from 205 breast invasive ductal carcinoma (IDC) patients who were treated at the Department of Breast Surgery, Key Laboratory of Breast Cancer in Shanghai, Fudan University Shanghai Cancer Center, from 2002 to 2006. Overall survival (OS) and disease-free survival (DFS) were compared.

**Results:**

In total, 152 patients (74.15%) were disease-free without relapse or metastasis, whereas 53 (25.85%) patients developed recurrence or metastasis. A significant positive correlation was observed between MyD88 and TLR4 expression (p<0.001). Patients with high expression were more likely to experience death and recurrence/metastasis events (p<0.05). Patients with low MyD88 or TLR4 expression levels had better DFS and OS than patients with high expression levels (log-rank test: p<0.001). Patients with low MyD88 and TLR4 expression levels had better DFS and OS than patients with high expression levels of either (log-rank test: p<0.001). In a multivariate analysis, high MyD88 expression was an independent predictive factor for decreased DFS (adjusted HR, 3.324; 95% CI, 1.663–6.641; p = 0.001) and OS (adjusted HR, 4.500; 95% CI, 1.546–13.098; p = 0.006).

**Conclusions:**

TLR4-MyD88 signaling pathway activation or MyD88 activation alone may be a risk factor for poor prognosis in breast cancer. Therefore, TLR4-MyD88 signaling pathway activation in tumor biology provides a novel potential target for breast cancer therapy.

## Introduction

Breast cancer is a major cause of death in women worldwide [1]. In China, one in six women will suffer from breast cancer during her lifetime [2], and within 20 years, breast cancer will become the most common cause of death in Chinese women. Tumor metastasis is the leading cause of death in patients with breast cancer after conventional treatment. However, it is difficult to predict the incidence of distant metastases due to the heterogeneity of breast cancer. Indeed, patients with the same histopathology and immunohistochemical characteristics may have completely different prognoses, highlighting the need for new therapeutic targets, especially for patients who are non-responsive or only partially responsive to conventional therapy [3], [4].

A dynamic association between breast cancer and the immune system is essential for its incidence, growth, and metastasis [5]. The inflammatory immune response caused is a double-edged sword; although it helps to fight against infection, the continued escalation of inflammation can facilitate tumor cell immune escape and negatively affect stability and health. Stimulation of chronic inflammation causes tumors to release many growth factors, resulting in an inflammatory microenvironment and promoting the occurrence and development of tumors. On one hand, tumor cells can secrete cytokines that attract inflammatory cells to migrate to tumor locations; on the other hand, inflammatory cells can also secrete proteolytic enzymes and cytokines that can stimulate the growth of tumor cells, promote the formation of local vascularization, and enhance the tumor capacity for local infiltration and metastasis [6]–[9].

As innate immune receptors, Toll-like receptors (TLRs) are crucial for both innate and subsequent adaptive immune responses [10]. Within the last decade, TLRs have received much interest in the field of cancer research due to their roles in tumor progression through factors released after TLR activation. To date, eleven types of TLRs have been found in humans, and thirteen TLR homologues have been detected in rats, mice, and fruit flies. TLRs are widely expressed in malignant tumor tissues. Human melanoma cells express TLR4 [11], and human cervical cancer cells and prostate cancer cells express TLR9. Human gastric cancer cells express TLR4, TLR5, and TLR9 [12], whereas human laryngeal cancer cells express TLR2, TLR3, and TLR4. Human neuroblastoma cells exhibit high levels of TLR4 expression. Human lung cancer cells express active TLR9, and mRNA expression of TLR1 to TLR10 has been detected in the metastatic human breast cancer cell line MDA-MB-231 and MCF-7 cells, which have low metastatic ability [13]. In addition, TLR4 has been studied widely in breast cancer [13], [14]. The results of these studies have shown that functional TLR expression occurs not only on immune cells but also on various tumor cells, playing an important role in the pathogenesis of tumors and the evasion of immune attack [15], [16].

TLRs on tumor tissues can be activated by the local presence of corresponding ligands, thus initiating epithelial transformation to a malignant phenotype and the secretion of a variety of cytokines that mediate the immune escape of tumor cells. This response stimulates the growth of tumor cells while strengthening the function of tumor cell infiltration [16], [17].

TLR4, the receptor for lipopolysaccharide (LPS), is unique in that it activates myeloid differentiation primary response 88 (MyD88)-dependent and MyD88-independent pathways [18]. MyD88, an adaptor protein that interacts with TLRs in the signaling pathway, can serve as a mediator for most TLRs except TLR3. In the MyD88-dependent pathway, the increased levels of IL-1 and IL-8 facilitate the immune response, killing invasive pathogens. In contrast, in the MyD88-independent pathway, TLR4 activate transcription via the TIR domain-containing adaptor [19]. Interestingly, TLR4 is the only TLR that can activate both of these pathways [10]. Rajput et al found that TLR4 activation promotes carcinogenesis and resistance to chemical treatments in breast cancer [13], [14], whereas blocking TLR4 activation can slow breast cancer growth and prolong survival [20]. However, the predominant pathway in TLR4 signal transduction remains unclear. Furthermore, little is known about the expression of MyD88 in breast cancer or its correlation with tumor development.

In the present study, we analyzed the expression of MyD88 and TLR4 in 205 cases of breast cancer and evaluated their correlation with the clinicopathological characteristics and prognoses of these patients.

## Materials and Methods

### Ethics

All of the samples were anonymously coded in accordance with local ethical guidelines. Written informed consent was provided by all participants, and all of the patient information was anonymized and de-identified prior to analysis. This study was approved by the Review Board of Fudan University Shanghai Cancer Center.

### Cell Lines

The human breast cancer cell lines MCF7, SKBR3, MDA-MB-231, and MDA-MB-468 were purchased from the American Type Culture Collection. All of these cell lines were cultured in Dulbecco’s Modified Eagle’s Medium (Gibco) supplemented with 10% (v/v) FBS (HyClone) at 37°C in a humidified incubator containing 5% CO2. The highly metastatic MDA-MB-231HM line, which has a high potential to metastasize to the lung, was established by our institute [21].

### Western Blotting

Cell lysates were clarified by centrifugation at 12,000 revolutions per minute. Equal amounts of proteins were separated by SDS-PAGE and transferred onto polyvinylidene difluoride (PVDF) membranes. The immunoblots were probed according to standard protocols with antibodies against MyD88 (Cell Signaling Technology, Boston, USA) and TLR4 (Abcam, Cambridge, USA). The quality of the loading and transfer was assessed by immunostaining with a β-actin antibody (Protein Tech Group, LA, USA). The immunoblots were developed using enhanced chemiluminescence reagent (Millipore, Massachusetts, USA), and images were captured using a Bio-Rad Molecular imager (Bio-Rad, California, USA). The expression intensity of the immunoblots was measured using Image Measure software.

### Patients and Specimens

We studied 205 stage I to III primary female breast cancer patients with IDC who were randomly selected from the database at the Department of Breast Surgery, Shanghai Cancer Center (no co-morbidities reported), from 2002 to 2006. Decisions regarding the therapeutic regimen were based on the Chinese Anti-Cancer Association guidelines for the diagnosis and treatment of breast cancer. These patients were followed regularly, and the clinical outcome was obtained and last updated in August 2013. The median follow-up time was 98 months (ranging from 2 to 144 months).

### Breast Cancer Tissue Microarray Construction

To construct the tissue microarray (TMA), tumor samples were collected prior to the initiation of cancer treatment, formalin-fixed and embedded in paraffin. A hematoxylin and eosin-stained section of each tumor block was used to define and mark representative tumor regions. The TMA sections were generated in the Department of Pathology at Fudan University Shanghai Cancer Center. Briefly, tissue cylinders with diameters of 2 mm were punched from the above regions and transferred to recipient array blocks using a Tissue Microarrayer. The TMA was composed of duplicate cores from different areas of the same tumor to compare the staining patterns.

### Immunohistochemistry

The expression of MyD88 and TLR4 was analyzed in TMAs of primary tumors. Briefly, the TMAs were dewaxed, hydrated, and washed, and the endogenous peroxidase activity was quenched. After high-pressure antigen retrieval, the TMAs were blocked and then incubated in a 1∶200 dilution of MyD88 or TLR4 antibody overnight at 4°C. Subsequently, the TMAs were rinsed and incubated with a working solution of horseradish peroxidase-conjugated secondary antibodies. After the samples were rinsed 3 times, staining was visualized using colorimetric detection with 3, 3-diaminobenzidine (DAB). The TMAs were counterstained with hematoxylin and subsequently dehydrated in graded alcohol and xylene.

### Evaluation of Immunohistochemical Variables

The evaluation of immunostaining was independently conducted by two experienced pathologists. The expression of MyD88 and TLR4 was scored according to the signal intensity and distribution. The staining intensity was ranked as follows: 1, weak; 2, moderate; and 3, intense. The staining distribution was determined in at least 5 areas of the slide and assigned a value from 0 to 4 as follows [22]: 0, <5%; 1, 5%–25%; 2, 25%–50%; 3, 50%–75%; and 4, >75%. Because of the heterogeneous characteristics of breast cancer, the predominant pattern was used for scoring. The percentage of staining distribution and the staining intensity were multiplied for each case. Tissues with an immunohistochemical score of 3 or less were considered to have low expression, and those with a score of 4 to 12 were considered to have high expression.

### Statistical Analysis

Disease-free survival (DFS) was defined as the time from the first diagnosis of breast cancer to the initial occurrence of disease relapse (local, regional, or distant). Overall survival (OS) was defined as the time between the date of the primary surgery and the date of death. Patients without any evidence of relapse or death were censored at the last date they were known to be alive. The probability of postoperative DFS and OS was derived from a Kaplan-Meier estimate, and the differences between the survival curves were compared using the log-rank test. MyD88 or TLR4 high-expressing tumors were compared with low-expressing tumors using the two-sided Pearson χ^2^ test. The effects of MyD88, TLR4, and other possible risk factors on DFS and OS were assessed by a Cox proportional hazards regression using a univariate or multivariate analysis with adjusted hazard ratios (HRs) and associated 95% confidence intervals (95% CI).

Statistical analyses were analyzed using the SPSS 18.0 software package (SPSS, Chicago, IL, USA). All P values were two sided, and p<0.05 was considered significant.

## Results

### Expression of MyD88 and TLR4 in Breast Cancer Cell Lines

**Figure 1:**
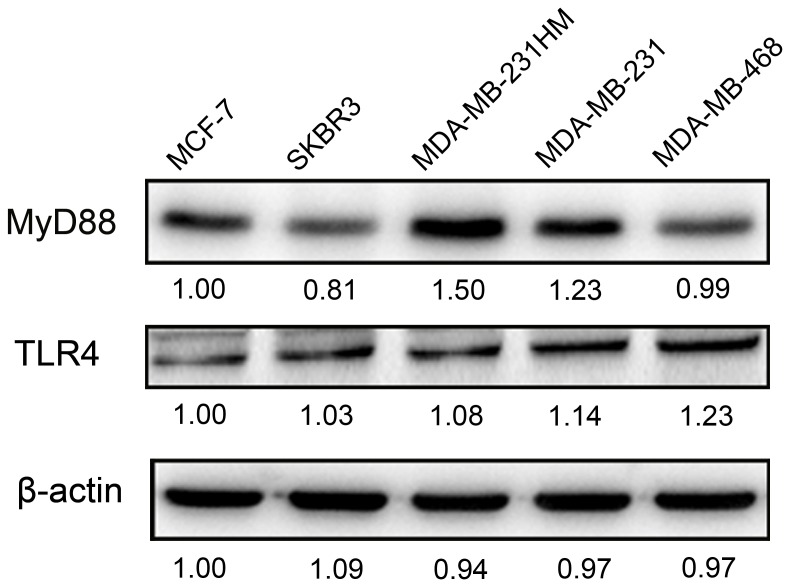
Protein expression of MyD88 and Toll-like receptor 4 in breast cancer cell lines.

We investigated the protein expression of MyD88 and TLR4 in MCF-7, SKBR3, MDA-MB-231HM, MDA-MB-231, and MDA-MB-468 cells by Western blotting. As shown in Figure 1, a high level of MyD88 and TLR4 protein expression was confirmed in the hormone-resistant breast cancer cell lines MDA-MB-231, MDA-MB-231HM, and MDA-MB-468 but not in the MCF-7 and SKBR3 cell lines.

### Patient Characteristics

All the tumor samples were patients who were diagnosed having IDC. The chemotherapy regimen for most of these patients was primarily based on anthracycline. The characteristics of the patients are presented in Table 1. During the follow-up, 152 patients (74.15%) were disease-free without relapse or metastasis, whereas 53 (25.85%) patients developed recurrence or metastasis. A total of 127 patients (61.95%) were lymph-node negative, and 77 patients were lymph-node positive (37.56%). The proportions were similar in different groups in terms of age, menopausal status and tumor size status.

**Table 1:** Clinical and histopathological features in breast cancer patients.

Characteristic	Number	Rate (%)
**Age, years**		
**≤50**	100	48.78%
**>50**	105	51.22%
**Unknown**	0	
**Menopausal status**		
**Premenopausal**	94	45.85%
**Postmenopausal**	111	54.15%
**Unknown**	0	
**Histological grade**		
**G 1–2**	153	74.63%
**G 3**	52	25.37%
**Unknown**	0	
**Tumor size**		
**≤5**	96	46.83%
**>5**	104	50.73%
**Unknown**	1	2.44%
**Lymph node**		
**Negative**	127	61.95%
**Positive**	77	37.56%
**Unknown**	1	0.49%
**Recurrence or metastasis**		
**No**	152	74.15%
**Yes**	53	25.85%
**Unknown**	0	

**Abbreviations**: G, Histological Grade.

### MyD88 Expression Was Positively Correlated with TLR4 Expression

**Figure 2:**
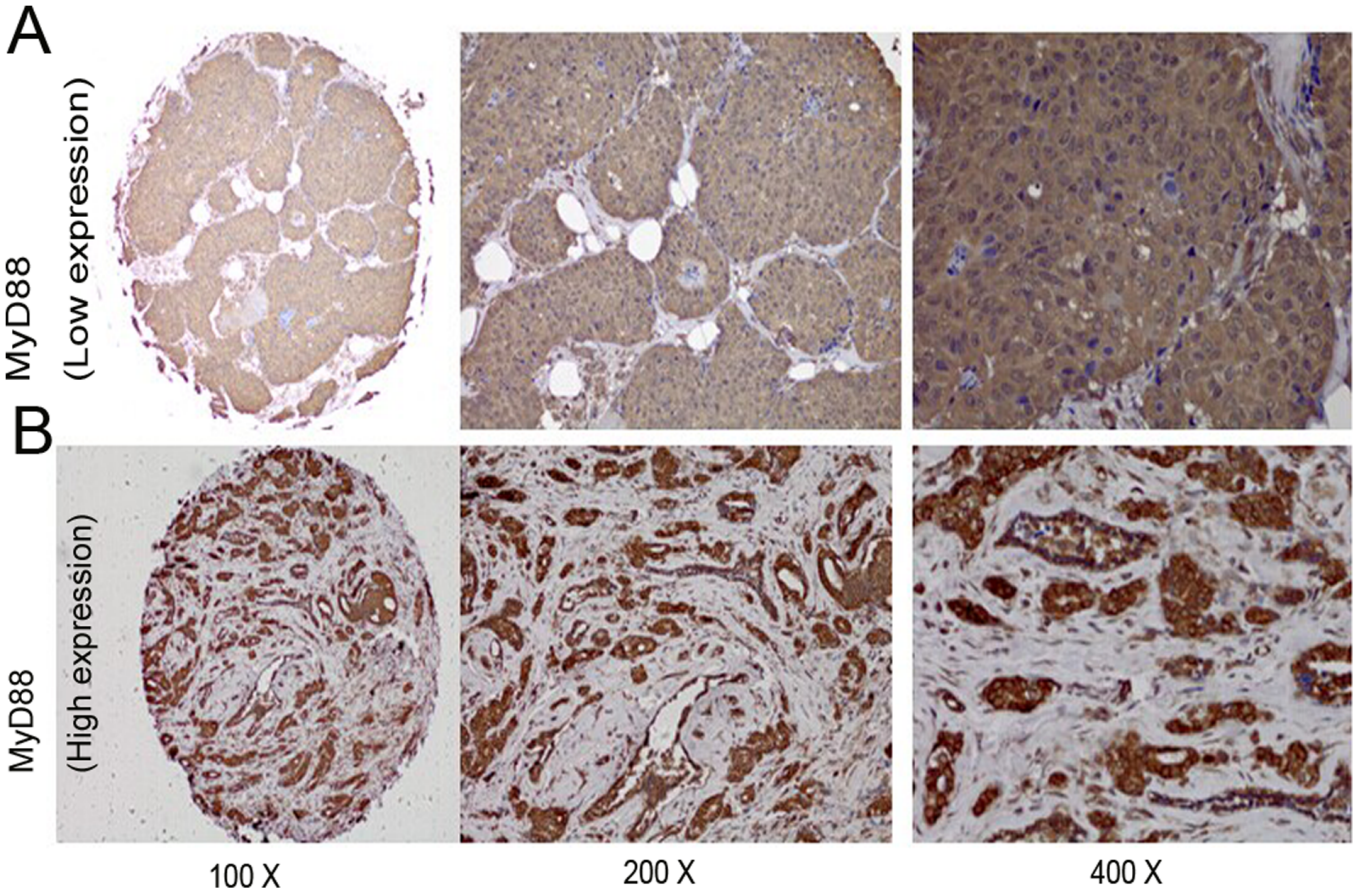
Representative MyD88 immunohistochemical staining in large (200× and 400× magnification) and small images (100× magnification). (A) Low immunostaining of MyD88 in invasive ductal breast carcinoma. (B) High immunostaining of MyD88 in invasive ductal breast carcinoma.

**Figure 3:**
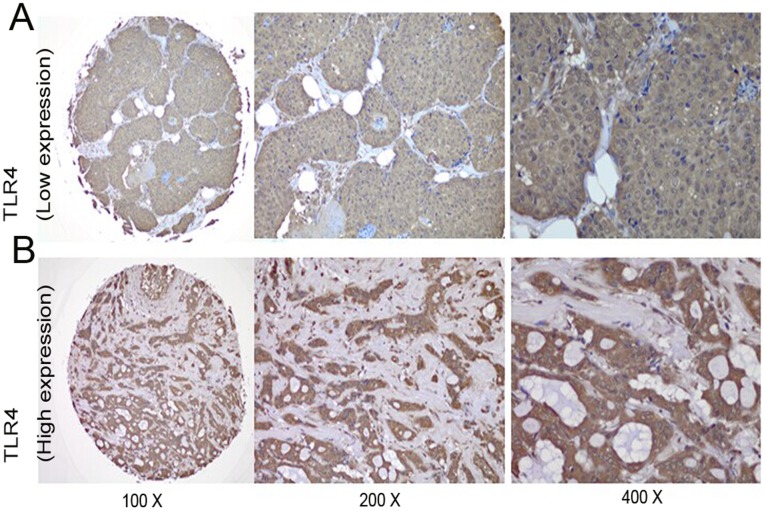
Representative TLR4 immunohistochemical staining in large (200× and 400× magnification) and small images (100× magnification). (A) Low immunostaining of TLR4 in invasive ductal breast carcinoma. (B) High immunostaining of TLR4 in invasive ductal breast carcinoma.

We then investigated the relationship between the expression levels of MyD88 and TLR4 in clinical breast cancer samples by immunohistochemical staining. Representative images of MyD88 and TLR4 are presented in Figure 2 and Figure 3, respectively. MyD88 staining was primarily observed in the cytoplasm and nuclei of tumor cells. High levels of MyD88 expression were detected in 89 of the 205 cases, whereas low expression levels were found in 116 cases. TLR4 was observed in the cytoplasm and cell membrane by immunostaining. High levels of TLR4 staining were observed in 94 of the 205 breast cancer patients, and low levels of staining were observed in the other 111 cases. A significant positive correlation was observed between the expression of MyD88 and TLR4 (p<0.001, Table 2).

**Table 2:** Correlation of the expression of MyD88 and TLR4.

	MyD88			
TLR4	Low	High	?^2^	P
**low**	85 (73.28%)	26 (29.21%)	**39.381**	**<0.001**
**high**	31(26.72%)	63 (70.79%)		

**Abbreviations**: MyD88, myeloid differentiation primary response 88; TLR4, Toll-like receptor 4;

**Bold values denote P≤0.05.**

### Correlations between MyD88 and TLR4 and Other Clinical and Pathological Parameters

We then investigated correlations between MyD88 and TLR4 expression and other clinical and pathological parameters (Table 3). The expression of MyD88 was associated with ER and PR status (p<0.001). TLR4 expression was associated with PR status (p = 0.015). Recurrence or metastasis occurred more frequently in patients with high MyD88 (p<0.001) and TLR4 (p = 0.006) expression levels. No significant associations were observed with other tumor parameters.

**Table 3:** Correlation of MyD88 and TLR4 with other clinical and pathological parameters.

Characteristic	MyD88 (low)	MyD88 (high)	?2	P	TLR4 (low)	TLR4 (high)	?2	P
**Age, years**			0.027	0.889			0.103	0.780
**≤50**	56	44			53	47		
**>50**	60	45			58	47		
**Unknown**	0	0			0	0		
**Menopausal status**			0.113	0.778			0.762	0.402
**Premenopausal**	52	42			54	40		
**Postmenopausal**	64	47			57	54		
**Unknown**	0	0			0	0		
**Histological grade**			2.053	0.195			1.034	0.337
**G 1–2**	91	62			86	67		
**G 3**	25	27			25	27		
**Unknown**	0	0			0	0		
**Tumor size**			0.665	0.480			3.079	0.092
**≤5**	57	39			58	38		
**>5**	58	50			52	56		
**Unknown**	1	0			1	0		
**Lymph node**			0.168	0.771				
**Negative**	73	54			73	54	1.715	0.196
**Positive**	42	35			37	40		
**Unknown**	1	0			1	0		
**ER**			**19.697**	**<0.001**			**4.092**	**0.058**
**Negative**	59	72			64	67		
**Positive**	57	17			47	27		
**Unknown**	0	0			0	0		
**PR**			**24.027**	**<0.001**			**6.341**	**0.015**
**Negative**	78	83			80	81		
**Positive**	38	4			30	12		
**Unknown**	0	2			1	1		
**HER-2**			3.159	0.085			2.173	0.154
**Negative**	64	60			62	62		
**Positive**	52	29			49	32		
**Recurrence or metastasis**			**14.892**	**<0.001**			**7.753**	**0.006**
**No**	98	54			91	61		
**Yes**	18	35			20	33		
**Unknown**	0	0			0	0		

**Abbreviations**: MyD88, myeloid differentiation primary response 88; TLR4, Toll-like receptor 4; G, Histological Grade; ER, estrogen receptor; PR, progesterone receptor; HER-2, human epidermal growth factor receptor 2;

**P-value was calculated with a two-sided χ2 test.**

**Bold values denote P≤0.05.**

### Elevated MyD88 and TLR4 Expression Was Associated with Poor DFS and OS

**Figure 4:**
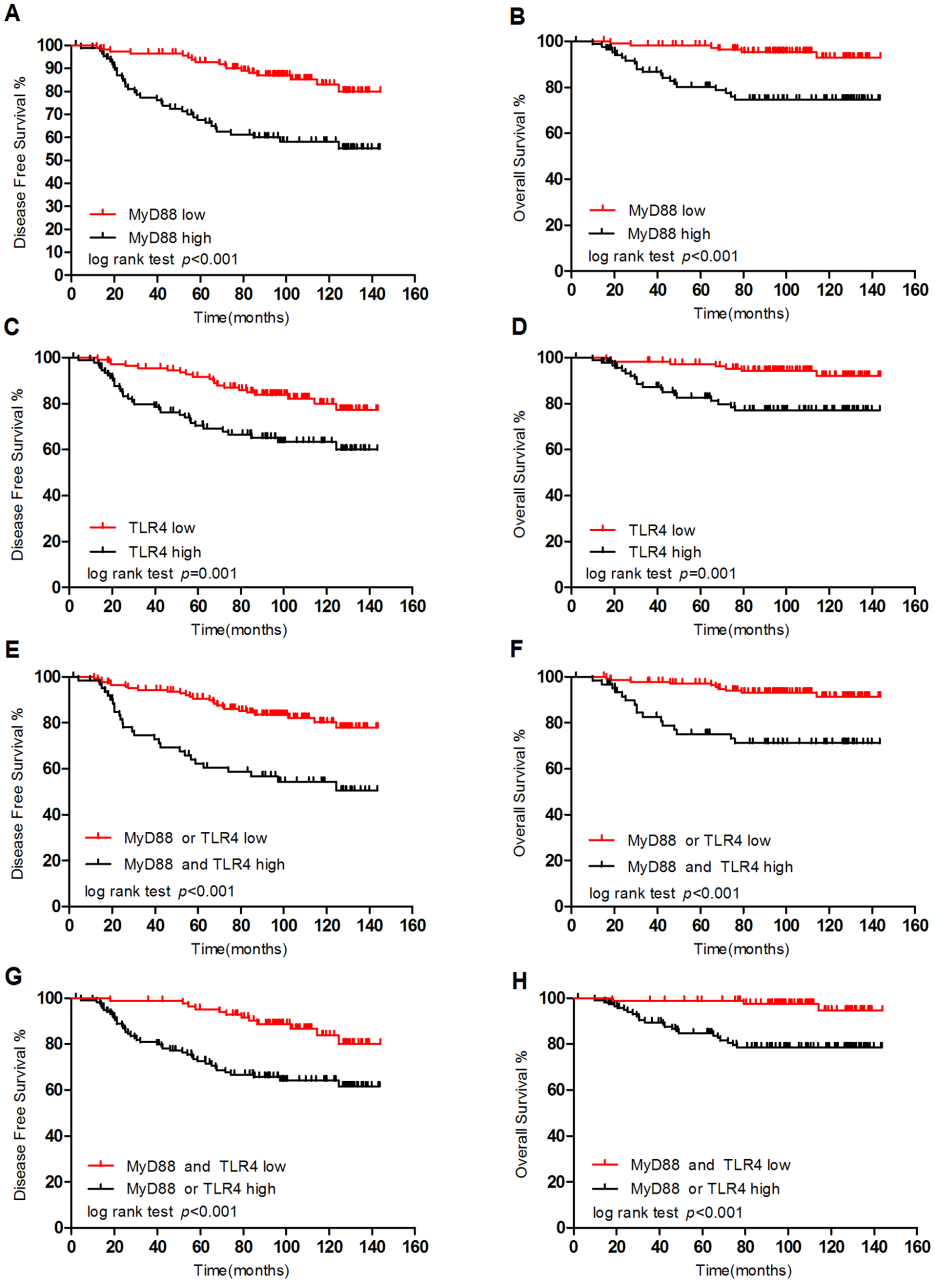
Kaplan-Meier curves based on MyD88 and TLR4 expression. (A), (B): Kaplan-Meier analysis estimates for disease-free survival and overall survival based on MyD88 status. (C), (D): Kaplan-Meier analysis estimates for disease-free survival and overall survival based on TLR4 status. (E), (G): Kaplan-Meier analysis estimates for disease-free survival based on MyD88 and TLR4 status. (F), (H): Kaplan-Meier analysis estimates for overall survival based on MyD88 and TLR4 status.

Using the log-rank test, significant differences were observed in the DFS and OS curves in terms of MyD88 expression (Figure 4A–B). Patients with low MyD88 expression levels had a better DFS than those with high expression levels (log-rank test: p<0.001). Differences in OS were also observed between patients with low and high expression levels of MyD88 (log-rank test: p<0.001). Figure 4C–D shows the survival curves according to TLR4 expression. The probability of DFS in TLR4 low-expressing patients was higher than that in TLR4 high-expressing patients (log-rank test: p = 0.001). A significant difference was observed in OS between low TLR4 expression and high TLR4 expression (log-rank test: p = 0.001). Patients with either low MyD88 or low TLR4 expression had higher DFS than patients with high expression of both MyD88 and TLR4 (log-rank test: p<0.001). A significant difference was also observed for OS (log-rank test: p<0.001). Furthermore, patients with a low expression level of MyD88 and TLR4 demonstrated improved DFS and OS compared to patients with a high expression level of either protein (log-rank test: p<0.001).

### Elevated Expression of MyD88 Was an Independent Predictive Factor for Reduced DFS and OS

A univariate analysis of the relationships between tumor characteristics and patient outcome indicated that the histological grade, lymph node status, and expression of MyD88 and TLR4 were significantly associated with DFS (p<0.05), whereas no significant prognostic values (p>0.05) were found with the other factors listed in Table 4. Menopausal status, histological grade, lymph node status, and expression of MyD88 and TLR4 were significantly correlated with OS (p<0.05); no correlations were found for the other factors (p>0.05) (Table 4).

**Table 4:** Univariate analysis of potential factors predicting disease-free survival and overall survival in breast cancer patients.

	Disease-free Survival		Overall Survival	
Variable	HR (95%CI)	P	HR (95%CI)	P
**Postmenopausal**	1.688 (0.953–2.989)	0.073	**3.778 (1.424–10.021)**	**0.008**
**Histological grade ≥3**	**1.799 (1.023–3.164)**	**0.042**	**2.209 (1.014–4.809)**	**0.046**
**Tumor size >5**	1.417 (0.817–2.457)	0.215	1.907 (0.850–4.279)	0.118
**Lymph-node positive**	**2.664 (1.529–4.641)**	**0.001**	**5.357 (2.250–12.755)**	**<0.001**
**ER**	0.892 (0.507–1.569)	0.692	0.731 (0.318–1.680)	0.460
**PR**	0.530 (0.239–1.175)	0.118	0.472 (0.142–1.572)	0.221
**HER2**	0.797 (0.453–1.430)	0.432	0.512 (0.215–1.218)	0.130
**MyD88**	**3.416 (1.912–6.103)**	**<0.001**	**5.268 (2.114–13.128)**	**<0.001**
**TLR4**	**2.422 (1.384–4.238)**	**0.002**	**3.916 (1.644–9.324)**	**0.002**

**Abbreviations**: MyD88, myeloid differentiation primary response 88; TLR4, Toll-like receptor 4; ER, estrogen receptor; PR, progesterone receptor; HER-2, human epidermal growth factor receptor 2; HR, hazard ratio; CI, confidence interval.

**P-value was calculated with a two-sided χ2 test.**

**Bold values denote P≤0.05.**

A Cox proportional hazard regression model was used to determine factors that were independent or joint predictors of DFS and OS. The multivariate analysis indicated that MyD88 status was an independent risk factor for DFS (adjusted HR, 3.324; 95% CI, 1.663–6.641; p = 0.001) and OS (adjusted HR, 4.500; 95% CI, 1.546–13.098; p = 0.006); lymph node status was also an independent risk factor for DFS (adjusted HR, 2.453; 95% CI, 1.388–4.336; p = 0.002) and OS (adjusted HR, 4.391; 95% CI, 1.790–10.771; p = 0.001) (Table 5); and postmenopausal status was also an independent risk factor for DFS (adjusted HR, 1.953; 95%CI, 1.056–3.614; p = 0.033) and OS (adjusted HR, 4.738; 95% CI, 1.721–13.046; p = 0.003).

**Table 5:** Multivariate analysis of potential factors predicting disease-free survival and overall survival in breast cancer patients.

	Disease-free Survival		Overall Survival	
Variable	HR (95%CI)	P	HR (95%CI)	P
**Postmenopausal**	**1.953 (1.056–3.614)**	**0.033**	**4.738 (1.721–13.046)**	**0.003**
**Histological grade ≥3**	1.414 (0.783–2.511)	0.250	1.409 (0.620–3.205)	0.413
**Tumor size >5**	1.541 (0.840–2.825)	0.162	2.055 (0.849–4.970)	0.110
**Lymph-node positive**	**2.453 (1.388–4.336)**	**0.002**	**4.391 (1.790–10.771)**	**0.001**
**MyD88**	**3.324 (1.663–6.641)**	**0.001**	**4.500 (1.546–13.098)**	**0.006**
**TLR4**	1.182 (0.601–2.325)	0.628	1.300 (0.449–3.763)	0.629

**Abbreviations**: MyD88, myeloid differentiation primary response 88; TLR4, Toll-like receptor 4; ER, estrogen receptor; PR, progesterone receptor; HER-2, human epidermal growth factor receptor 2; HR, hazard ratio; CI, confidence interval.

**P-value was calculated with a two-sided χ2 test.**

**Bold values denote P≤0.05.**

## Discussion

The TLR4 signaling pathway includes MyD88-dependent and MyD88-pathways [18]. These pathways can cause chronic inflammation, which stimulates the release of growth cytokines by tumors, resulting in an inflammatory environment and promoting the occurrence and development of tumors. It is unknown which pathway plays the most important role in breast cancer, and little is known about the expression of MyD88 in breast cancer and its correlation with TLR4 in tumor prognosis. This is the first study to demonstrate the expression of MyD88 and TLR4 in 205 cases of IDC and evaluate their correlation with clinicopathologic characteristics and prognosis. IDC is the most common histological subtype of invasive breast cancer, representing more than two-thirds of breast cancer patients [4], . Because several studies have compared IDC and other types of breast carcinomas in Chinese women [24], [25], we included only IDC in our study and focused on the clinical significance of TLR4-MyD88 markers in IDC. Excluding lymph node status, the proportions of the patients were similar with respect to age, menopausal status and tumor size status; 77 (37.56%) patients were lymph-node positive, which is consistent with observations from previous studies [26], [27].

A significant positive correlation was observed between MyD88 and TLR4 immunostaining in the tissue samples of the patients (p<0.001), which is consistent with the results obtained using breast cancer cells lines (Figure 1). This finding indicated that activation of the TLR4 signaling pathway in breast cancer primarily occurs via a MyD88-dependent (not MyD88-independent) pathway. Subsequently, a high expression level of MyD88 and TLR4 was associated with poor DFS (log-rank test: p<0.001, p = 0.001, respectively) and OS (log-rank test: p<0.001, p = 0.001, respectively). Consistent with our results, TLR4 activation has been reported to accelerate breast cancer growth and reduce survival [13], [20]. However, the biological function of MyD88 in breast cancer has not been well defined. Thus, MyD88 expression may be helpful for predicting the prognosis of breast cancer patients. The role of TLR/MyD88 pathway activation in breast cancer has been controversial. Most studies have indicated that activation of the TLR/MyD88 pathway is related to poor prognosis [28], [29]; however, this pathway can also suppress cell proliferation and tumor growth [30]. Thus, we next analyzed the prognostic effect of TLR4-MyD88 activation in breast cancer. Consistent with most studies, high expression levels of MyD88 and TLR4 were related to poor DFS (log-rank test: p<0.001) and OS (log-rank test: p<0.001). In addition, low expression levels of MyD88 and TLR4 predicated improved DFS and OS compared with high expression levels of either protein (log-rank test: p<0.001), indicating that TLR4 and MyD88 were related to decreased DFS and OS.

We next sought to determine which factors were independent and yielded greater prognostic value. When the risk factors predicting DFS and OS were considered individually, histological grade and lymph node status remained significant (p<0.001), which is consistent with previous studies [26], [27], [31]. The expression of MyD88 and TLR4 appeared to be associated with poor DFS and OS rates (p<0.05). After adjusting for all of the risk factors, only histological grade and MyD88 remained independent risk factors. Thus, we can conclude that MyD88 is more important than TLR4 in TLR4-MyD88 signaling activation. The activation of TLRs expressed on breast cancer cells may result in profound consequences for tumor growth through MyD88.

We confirmed that MyD88 and TLR4 were highly expressed in hormone receptor-negative breast cancer cell lines. Thus, to analyze the distribution of MyD88 and TLR4 in breast cancer patients of different characteristic groups, we found that the expression of MyD88 was associated with ER and PR status (p<0.001) and that the expression of TLR4 was associated with PR status (p = 0.015), indicating that the expression of MyD88 and TLR4 may play an important role in breast cancer, especially in triple-negative breast cancer (TNBC). Because TNBC predicts an increase in mortality compared with other subtypes of breast cancer [23], [32], [33], clinical outcomes may improve if the risk factors that confer a poor prognosis in TNBC can be controlled. Our work provides new insight into the development/progression of inflammation and breast cancer, suggesting that activation of the TLR4-MyD88 signaling pathway or MyD88 alone may be risk factors for a poor breast cancer prognosis and may be novel targets for the development of biomodulators.

This study has significant limitations. TLR4 has been shown to be a potential receptor of paclitaxel, but the chemotherapeutic regimen for most of the patients in the present study was primarily based on anthracycline. Thus, the effect of the TLR4-MyD88 signaling pathway in patients treated with paclitaxel could not be determined. However, we did confirm that MyD88 and TLR4 were positively associated with each other, especially in TNBC patients.

## Conclusion

Chronic inflammation is often related to the occurrence and growth of various malignancies [34]. TLRs are the main innate immune pattern recognition receptors, and many researchers have closely investigated the molecular mechanism of TLR4, which promotes tumorigenesis, invasion, metastasis, recurrence, and drug resistance in breast cancer [13], [14], [20]. The present study focused on TLR4 and its prognostic value in cancer therapy. Few studies have investigated MyD88, an important adaptor protein of TLR4, in breast cancer. Thus, we assessed the expression of MyD88 and TLR4 in 205 breast cancer patients and confirmed that MyD88 and TLR4 expression was correlated with poor DFS and OS. MyD88 is an independent risk factor for poor prognosis, and the expression of MyD88 and TLR4 may play an important role in TNBC patients. These findings suggest that activation of the TLR4-MyD88 signaling pathway or MyD88 alone may be a risk factor for poor prognosis in breast cancer. Accordingly, the function of the TLR4-MyD88 pathway in tumor biology provides a new potential target for breast cancer therapy.
